# Maritime Spatial Planning supported by systematic site selection: Applying Marxan for offshore wind power in the western Baltic Sea

**DOI:** 10.1371/journal.pone.0194362

**Published:** 2018-03-15

**Authors:** Cordula Göke, Karsten Dahl, Christian Mohn

**Affiliations:** Department of Bioscience, Aarhus University, Roskilde, Denmark; University of Waikato, NEW ZEALAND

## Abstract

The development of offshore wind energy and other competing interests in sea space are a major incentive for designating marine and coastal areas for specific human activities. Maritime Spatial Planning (MSP) considers human activities at sea in a more integrated way by analysing and designating spatial and temporal distributions of human activities based on ecological, economic and social targets. However, specific tools supporting spatial decisions at sea incorporating all relevant sectors are rarely adopted. The decision support tool Marxan is traditionally used for systematic selection and designation of nature protection and conservation areas. In this study, Marxan was applied as a support tool to identify suitable sites for offshore wind power in the pilot area Pomeranian Bight / Arkona Basin in the western Baltic Sea. The software was successfully tested and scenarios were developed that support the sites indicated in existing national plans, but also show options for alternative developments of offshore wind power in the Pomeranian Bight / Arkona Basin area.

## Introduction

The Baltic Sea is a semi-enclosed marginal sea of the North Atlantic and one of the largest brackish water bodies in the world. There are 9 bordering countries with strong ecological, social and economic interests in Baltic Sea goods and services including environmental protection, shipping, fisheries, offshore energy and tourism. As a consequence, the Baltic Sea is one of the most used and ecologically threatened, but also one of best studied and regulated maritime areas worldwide [1–2].

Maritime Spatial Planning (MSP) has been identified as a useful concept by the European Union member states for a sustainable maritime development, integrating EU’s blue growth strategy with existing directives aiming to protect marine habitats and species, water quality and the marine environment in general [3–5]. The MSP Directive adopted by the European Union in 2014 has identified the energy sectors at sea, maritime transport and fisheries and aquaculture sectors as key planning objectives [6].Under this directive, the aim of spatial planning is to reduce conflicts between different interests in sea space, create synergies between activities, encourage cross-border cooperation and at the same time protect the environment. There has been only limited practical experience of MSP in the Baltic Sea to date, but current EU Maritime Policy actively seeks the implementation of a common framework for integrated MSP within all European seas [6]. The Helsinki commission (HELCOM) and the intergovernmental multilateral co-operation of the Baltic Sea Region in spatial planning and development VASAB (Visions and Strategies around the Baltic Sea) recently developed a roadmap and associated guideline for an ecosystem-based approach to MSP for the Baltic Sea [7–8].

It is a fundamental aspect of MSP to meet requirements set by other EU directives and legal acts for the improvement of the ecological status and water quality. Thus, maritime spatial planners aim to balance different requests for sea space and maintain or enhance a healthy marine environment at the same time. This process involves a variety of approaches, methods and practical software tools. Decision support software is widely accepted and used as a tool in systematic conservation planning to find cost-efficient zones for protecting biodiversity and meeting other important ecological targets (for an overview see [9]).

**Fig 1:**
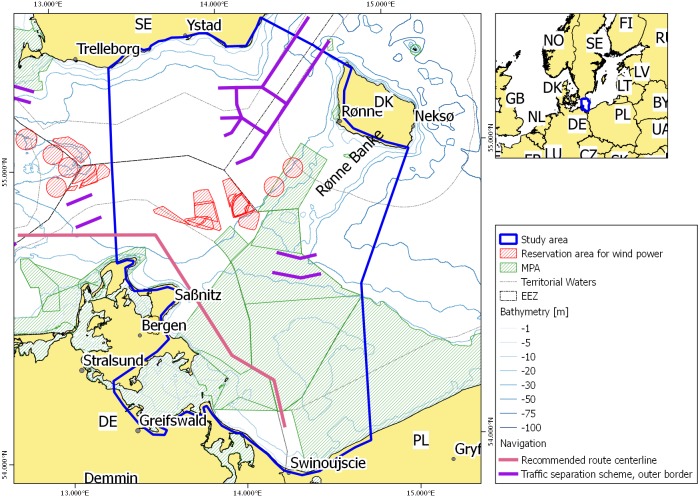
Overview over the case study area Pomerian Bight / Arkona Basin in 2011 with Natura 2000 areas, shipping routes (recommended route, separation schemes) and reservation areas for offshore wind farms. The EEZ border between Denmark and Poland is not drawn due to its unclear legal status. DE–Germany, DK–Denmark, PL–Poland, SE–Sweden.

In this study, we employ the widely used conservation planning software Marxan [10–12]. Marxan is a decision support tool using an optimization algorithm for site selection and is designed to find the most cost efficient suggestions for suitable marine conservation areas which meet a number of ecological, social and economic objectives. This study was conducted as part of the EU Interreg project BaltSeaPlan (www.baltseaplan.eu) and was integrated into a planning process over two years, where a team of experts with various professional backgrounds developed a pilot transboundary plan for the study area Arkona Basin/Pomeranian Bight in the western Baltic Sea (Fig 1). This process took place outside the formal MSP process, but involved the responsible authorities from Germany and experts from Poland, Germany and Sweden. In addition to generating a spatial plan that supports sustainable offshore wind development, this pilot study is also a test case for developing MSP across national borders and using decision support tools [13]. Hence, the main objective of applying Marxan was (alongside the concrete suggestion of suitable sites) to test the usefulness and reliability of a systematic decision support system for a specific MSP challenge other than nature conservation. In particular, Marxan has been set up to analyse the potential for offshore wind power in the study area for different cost and target scenarios.

## Methods and data

### Study area and the MSP process

The Pomeranian Bight/Arkona Basin covers an area of approximately 14,100 km^2^ and is situated in the western Baltic Sea and spans territorial waters and Exclusive Economic Zones (EEZs) of Denmark, Sweden, Poland and Germany (Fig 1). Different and sometimes conflicting interests compete for the limited space. Major commercial shipping routes with heavy ship traffic transit the area in the East-West direction. In addition, ferry lines are operating in the North-South direction connecting Sweden and Bornholm, Sweden and Germany and Sweden and Poland respectively. The southern part of the study area is especially important for seabirds and accommodates numerous habitats. Thus, large areas are designated Natura 2000 areas protecting birds, marine mammals or habitats listed in the Birds or Habitats Directive. Tourism and industrial infrastructure and activities (ports and harbours) dominate the coastal zone. Sand and gravel extraction, pipelines and submarine cables are other examples of competing uses that have an impact on the seabed.

The extension of the offshore wind energy sector is driven by future demand of renewable energy and general commitments to reduce the emissions of greenhouse gases. As a consequence, claims for offshore wind energy are growing and increasingly in conflict with other interests such as buffer zones for maritime safety (shipping, cables and pipelines), tourism, migration routes (seabirds, other migratory marine species) and fishery. At the time of this study, spatial reservation for two offshore wind parks of 200 MW each were made in Danish waters near Rønne Banke (Fig 1 and [14]). High investment costs were foreseen for the two sites due to large water depths and distance from shore. Planners had also to consider the importance of Rønne Banke for harbour porpoise and seals, as well as for migrant birds in winter and spring. The areal reservation at Rønne Banke was partly overlapping with the later appointment of a Natura 2000 area in 2010 and subsequently changed after completion of this study. Offshore wind development is more advanced in the German part of the study area. Several applications within the German EEZ and the territorial waters of the federal state of Mecklenburg-Western Pomerania had already passed the planning and permitting stage at the time of this study (2012) and construction of one site is presently ongoing. At the same time, evaluation of suitable sites for offshore wind development was carried out in Poland, whereas Sweden did not plan development of offshore wind parks in the study area (Fig 1).

### Marxan setup

The study area was subdivided into a total of 14481 hexagonal planning units (PUs) of 1 km^2^ in size to facilitate sufficient spatial resolution of competing uses, environmental components and the irregular shape of the study area. Marxan is a tool for optimizing a specific objective based on user defined targets (Eq 1). In this study, the objective function was set to meet the offshore wind planning targets and avoiding conflicts with important ecological features, reducing conflicts with other human uses and minimizing construction costs. Thus, the objective function for all PUs is to minimize the following score [15]:

$$\sum_{PUs}Cost+(BLM*\sum_{PUs}Boundary)+(\sum_{Target\;Features}Penalty*FPF)$$ (1)

PUs = selected Planning Units
Cost = Cost for ecological features, human uses and construction costs
Boundary = Boundary length of the selected PU
BLM (Boundary Length Modifier) = weight to control the aggregation of the selected PUs
Target Feature = Potential for offshore energy production
Penalty = Difference between target set and selected amount
FPF (Feature Penalty Factor) = Penalty factor for not reaching the target for the feature. FPF affects the likelihood of reaching a specific target per target feature

**Fig 2:**
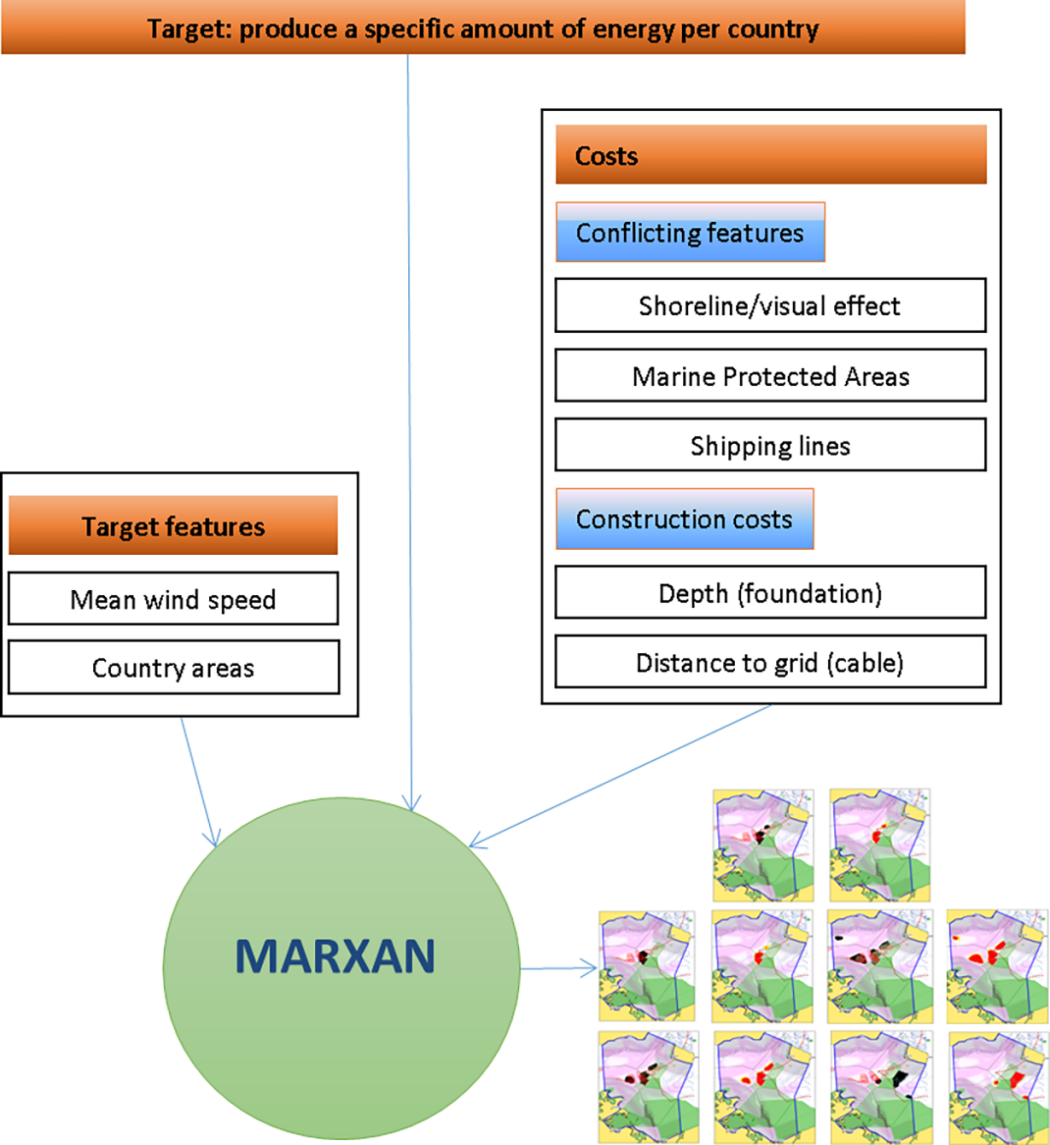
Target and data layers that have been included in Marxan to find suitable solutions for offshore wind power in the western Baltic Sea.

The main target was to find suitable sites for producing a specific amount of wind energy per country based on wind availability, costs for foundation and cable connections. The setup for offshore wind power was based on previous experiences published in the Danish strategy for future offshore wind power sites (English summary:[14], complete study in Danish: [16]). Costs were applied without compensating for future developments or differences between the countries involved in the study. Conflicting features are treated as costs and include the visual disturbance of the tourism, conflicts with shipping and ecological features (Fig 2).

The Danish Energy Authority concludes that different wind mill sizes and possible spacing for them result in similar technical production capacity per area [16]. 44 km^2^ were chosen for a 200 MW park. Whereas the target features are kept as separate layers and their individual influence is controlled by the FPF, the cost components are summed up per PU and need to be properly set in relation to each other. The input data was therefore weighted to represent 200 MW wind parks at mean wind speed in the area. The non-monetary costs were set into relation to the monetary costs to achieve an adequate impact.

The Marxan algorithm utilizes iteration to optimize Eq 1. The number of iterations can be varied mainly to avoid unnecessary processing time while ensuring to consistently produce similar solutions close to the optimum. While the iteration process is the basis for one optimized solution, it is expected that there are more than one solution within an area. Because the iteration steps include a random selection of planning units, the result of the iteration can be different for every run. Thus, every scenario was iterated several times, which provides the selection frequency for the single planning units. A selection frequency of > 50% is considered as a significant solution [17–18].The so called “best solution” is the solution for the run which minimizes Eq 1 most [19]. The number of iterations was set to 10^6^ and the number of repetitions to 100 for each scenario.

### Data description

The country targets for Germany are defined as areas and for Denmark as areas as well as expected production within the area (personal communication, [16]). Thus the respective country’s area (territorial waters and EEZ) is the first target feature and the target is defined as percentage of the area. The mean wind speeds act as the most important target layer to find solutions suitable for high energy production.

**Fig 3:**
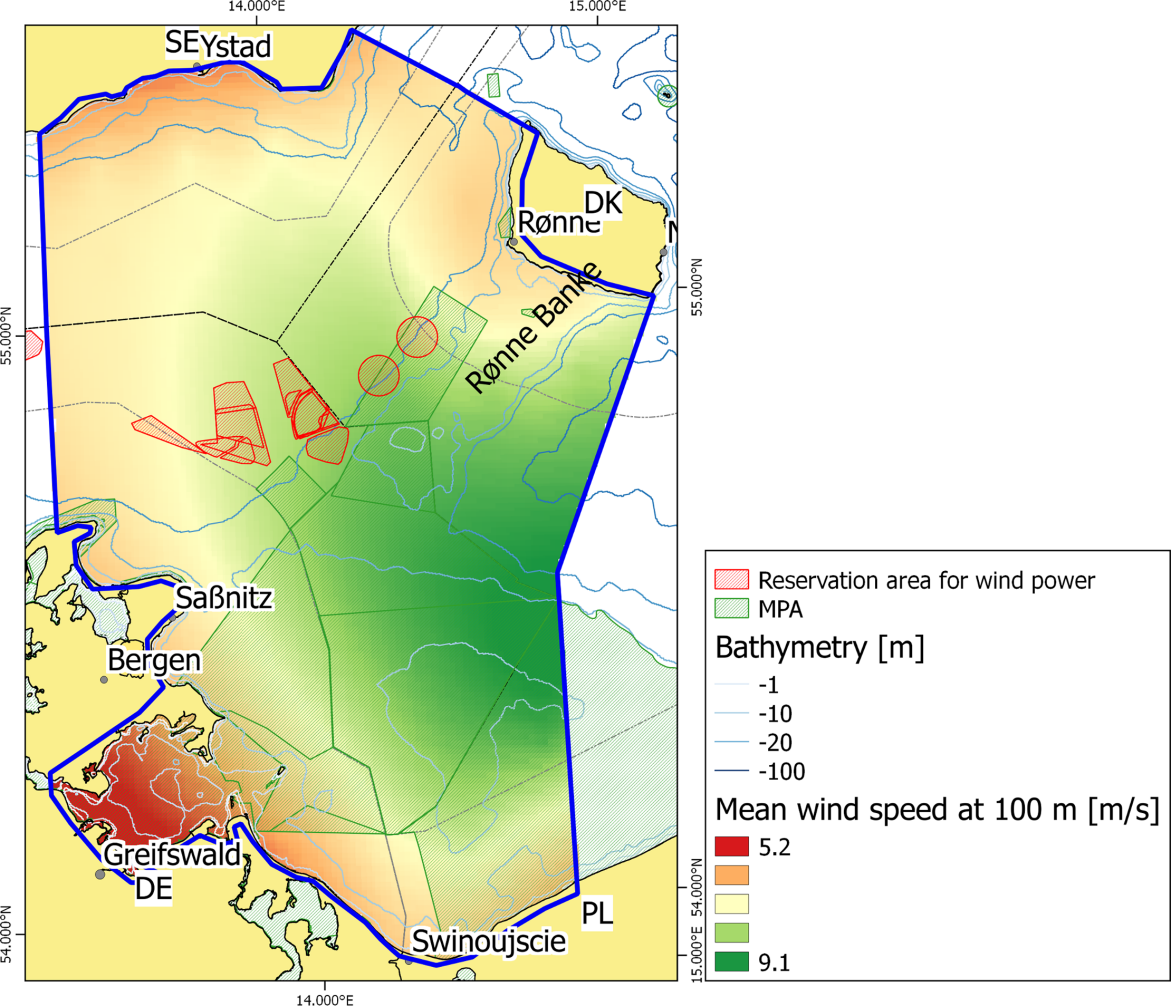
Average wind speed at 100 m height for the years 1998–2007. DE–Germany, DK–Denmark, PL–Poland, SE–Sweden.

Mean wind speeds for the period 1998 to 2007 at a height of 100 m (typical turbine height at the time of the study) were obtained from a dataset provided by the Interreg IIIa project POWER (PL/LT/RU). The wind distribution had been modelled with the UMPL (Unified Model for PoLand area) model v4.5 with a resolution of 9 nautical miles (approximately 17 km) and smoothed to a higher technical resolution. The smoothing does not take into account coastal influences on the wind distribution (Fig 3). The Danish Energy Authority estimates wind energy production on the basis of existing offshore wind parks [14]. Only two data points were available for estimating the energy output. It is thus based on the energy production of 3900 and 4300 full load hours for mean wind speeds 9.4 ms^-1^ and 10.3 ms^-1^ respectively and the possible energy production was estimated as a linear relation. The target layers were normalized so that they have a maximum value of 165 (Table 1).

**Table 1:** Value ranges per PU for the target layers. For the features divided into classes the value still varies for those PU that are only partly covered by the feature.

	Wind	Denmark	Germany
min	0	0	0
max	165	165	165
type of function	Linear	1 class	1 class

The cost layer in Marxan comprises of construction costs as well as conflicting features. Only construction costs that vary due to geographical features between different sites have been taken into consideration. They include distances to the electrical grid (cable connection costs) and bathymetry (foundation costs). The foundation costs were again estimated based on existing facilities [14]. The bathymetry was taken from the BALANCE dataset [20]. Missing information on existing or planned land-sea electric connection points was replaced by the distance to land, including the island of Rügen. For distances > 50 km, converter stations to high voltage direct current (HVDC) are needed which substantially increase the connection costs for a wind park. Due to a lack of information about the actual construction of converter stations, a constant factor of 30 for all distances > 50 km was added to the connection costs.

While the foundation costs are defined per MW, the cable connection to the grid is relevant for one or more complete parks. To set these two cost factors into relation to each other, a park size of 44 km^2^ or 200 MW was assumed [16].

There are three potential major conflicting issues in the study area: (1) Maintaining functional maritime transport, ferry connections and relevant ports, (2) securing the scenic value for maritime and coastal tourism, and (3) protecting sensitive and valuable natural environments including Natura 2000 areas, important seabird wintering areas and spawning areas of Baltic herring.

The requirements for shipping routes are described by data from AIS (Automated Identification System) vessel tracking and IMO (International Maritime Authority) regulations. The weighting of single routes, e.g. ferry connections with relatively low AIS vessel density, was adjusted for those routes where the traffic density does not describe the route’s importance (Table 2). A new IMO separation scheme for the area southwest of Bornholm was planned but not introduced yet. Thus, we replaced the existing used routes as indicated by AIS data with the most sensible connection from existing routes to the planned separation scheme. The routes were surrounded by a 2 nm wide buffer zone. Maintaining undisturbed views for coastal tourism was accompanied by strict regulations at that time both in Germany and Denmark. In Germany windmills closer to the coast than 20 km were rejected (personal communication). A detailed Danish analysis concluded that at 20 km distance 125 m high turbines would occasionally be visible from the coast, with only a very limited number of days each year when visibility exceeds 19 km [14]. It was agreed in the expert team to use the same approach for all countries, so a buffer to 20 km from coast was given the value 50. Natura 2000 sites were treated as in conflict with windparks.

**Table 2:** Value ranges per planning unit (PU) for the layers that compose the cost values. For the features divided into classes the value still varies for those PU that are only partly covered by the feature.

	Foundation	Connection to grid	Connection length > 50 km	Shipping	MPA	View
Maximum values per PU	98	7.6	30	30	50	50
Type of function	exponential	linear	single class	3 classes (6, 24, 30), decreasing in security buffer	single class	single class
Total (Sum over all PUs)	323065	46349	64590	158167	297550	386200

### Selection of scenarios

Four scenarios with different target and cost settings were analysed with Marxan (Table 3). In this comparison, the targets are represented as areas. For the main target layer based on the mean wind speed, the targets are calculated as described in the methods. Scenario 1 is the reference scenario based on the real case planning status at the time of this study. The influence of an increasing wind energy demand on site selection has been estimated, using double and threefold targets (scenarios 2, 3). Scenario 4 aims to demonstrate the differences in selecting suitable areas when costs for cable connections to existing land-based electrical are neglected assuming the possibility to connect to a supergrid. Individual country targets were only considered in Scenario 1 because it has the objective to provide settings comparable to the planning process leading to the individual national plans for offshore wind power in the case study area at that time. In all other scenarios the targets were not divided into country targets to investigate the optimal solution for offshore wind power development within an international transboundary area. The idea of a supergrid was introduced in scenario 4 to demonstrate the influence of both the coordinated site selection for wind power and the coordination of the effort to build cable connections.

**Table 3:** Overview of Marxan scenarios based on differences between target features and investment costs.

	Scenario 1	Scenario 2	Scenario 3	Scenario 4
Target	218 km^2^ wind parks separated into country targets for Germany (130 km^2^) and Denmark (88 km^2^)	436 km^2^ wind parks without separation into country targets	654 km^2^ wind parks without separation into country targets	436 km^2^ wind parks without separation into country targets
Costs	Foundation and connection to grid	Foundation and connection to grid	Foundation and connection to grid	Foundation

## Results

**Fig 4:**
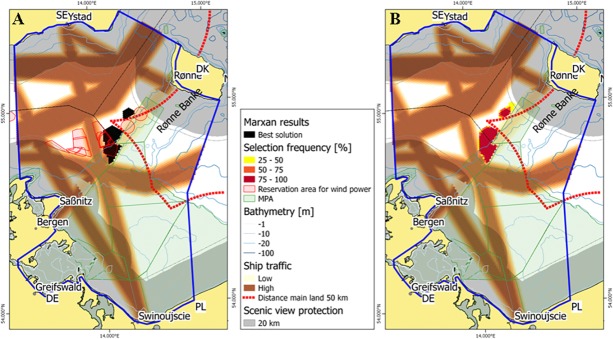
Marxan selection of most suitable offshore wind park sites for scenario 1 target 218 km^2^. (A) Best solutions by Marxan (black) compared to sites considered by national plans (red hatches); (B) selection frequency from different Marxan runs. DE–Germany, DK–Denmark, PL–Poland, SE–Sweden.

The main objective of reference scenario 1 was to test the capability of Marxan to reproduce suitable offshore wind sites similar to the existing national plans at the time of this study. A total wind park area of 218 km^2^ separated into country targets for Germany (130 km^2^) and Denmark (88 km^2^) was prescribed as the main target feature (German target: pers. communication, Danish target: [14]). Corresponding country targets for Poland and Sweden were not set at the time. The methodology and data basis as described above was agreed in the planning group and differs from the approach in Denmark and Germany. The locations of suitable sites selected by Marxan are presented in Fig 4A and are compared with corresponding sites considered by existing national plans. Marxan selections did not exactly match the location of sites in the national plans: Areas of high wind intensity southwest of the island of Bornholm and at distances to the grid of less than 50 km were predicted as the best suitable sites for offshore wind energy development by Marxan. Comparing the best solution by Marxan and the selection frequency, there is little variation in the results (Fig 4B). For the German side, the actual planned size as communicated by the planning authorities was 130 km^2^, but the reservation areas as designated in the maps stretch over 283 km^2^.

Even though the considerations leading to the Danish strategy were very well documented, it is not clear, how exactly the designated areas were selected. The differences can therefore be attributed to different experimental approaches and changes in the situation and data between the development of the national strategies (for Denmark finalized in 2007) and this study. For example, existing Natura 2000 areas were excluded both by the Danish Energy Authority and Marxan as potential sites for wind energy development [16]. However, the Danish offshore wind energy planning process was conducted before the introduction of the Natura 2000 area “Adler Grund” and Rønne Banke” in 2010. Thus, the areal reservation for wind energy sites in the Danish national plan overlaps with the Natura 2000 area.

**Fig 5:**
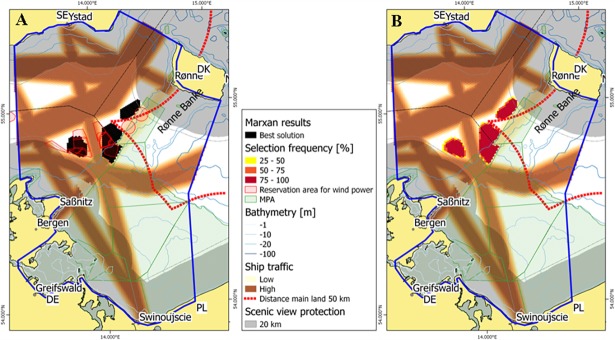
Marxan selection of most suitable offshore wind park sites for scenario 2 based on a twofold increase of energy production (436 km^2^). (A) Best solutions by Marxan (black) compared to sites considered by national plans (red hatches); (B) selection frequency from different Marxan runs. DE–Germany, DK–Denmark, PL–Poland, SE–Sweden.

**Fig 6:**
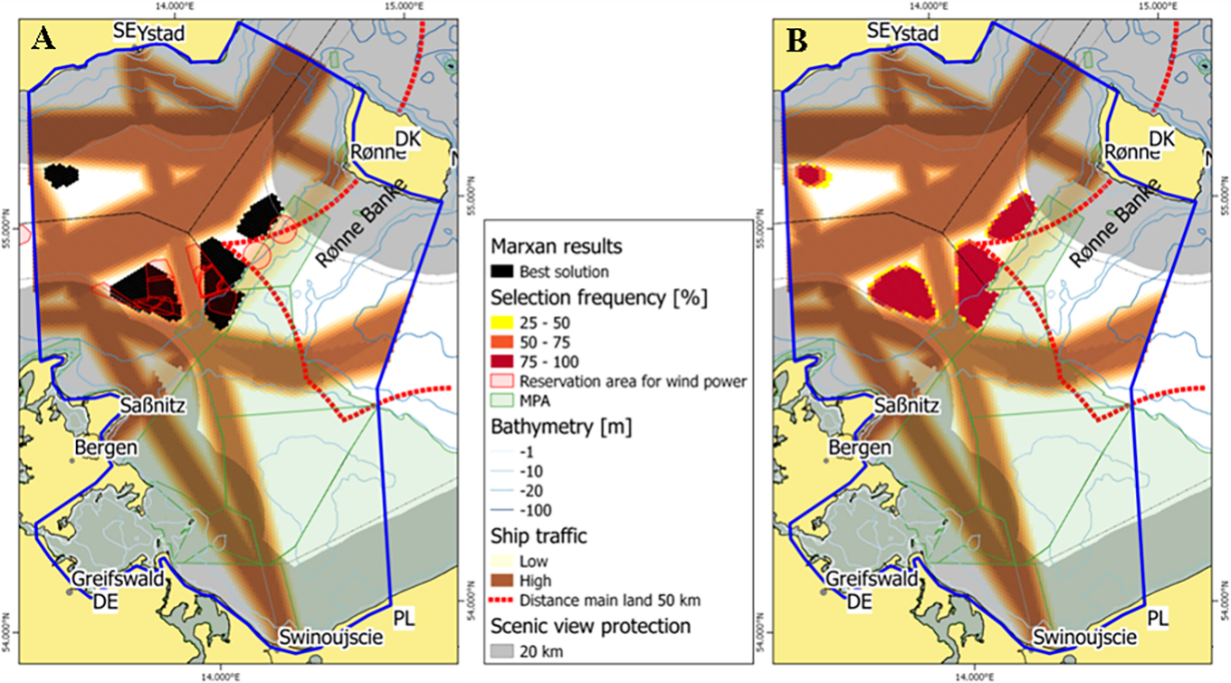
Marxan selection of most suitable offshore wind park sites for scenario 3 based on a threefold increase in energy production (654 km^2^). (A) Best solutions by Marxan (black) compared to sites considered by national plans (red hatches); (B) selection frequency from different Marxan runs. DE–Germany, DK–Denmark, PL–Poland, SE—Sweden.

Increased wind energy target features have been tested in scenarios 2 and 3. These targets include a twofold (scenario 2) and threefold (scenario 3) increase of wind energy production expressed as area, in comparison with the targets set in scenario 1 (Table 3). Individual country targets were not considered. A total target of 436 km^2^ was prescribed for scenario 2. The resulting sites selected by Marxan again represent the best solution and largely agree with the location of two large and approved sites in the German national plan for Mecklenburg–Western Pomerania (Fig 5A and 5B). However, the third and smaller site situated close to the German territorial border was not chosen by Marxan due to its proximity to the security buffer zone of important shipping routes and more restrictive Marxan settings defining a larger area for scenic view protection (Fig 5A). The threefold increase in the initial energy production target in scenario 3 produced a comparable Marxan selection of potentially suitable sites with two exceptions: Firstly, the selected sites partly extended towards the location of the smaller approved site at the German territorial border. Secondly, a small site in shallower Swedish coastal waters was selected (Fig 6A and 6B).

**Fig 7:**
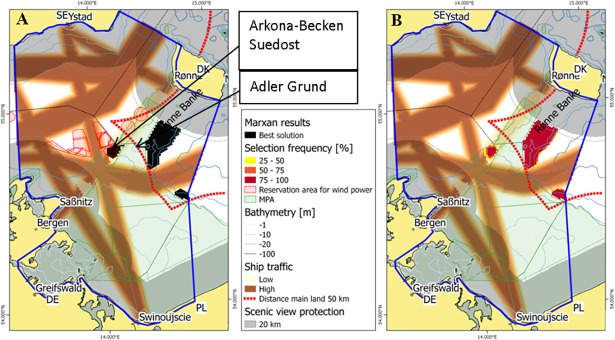
Marxan selection of most suitable offshore wind park sites for scenario 4 based on a twofold increase of energy production (436 km^2^). Grid connection costs were not considered. (A) Best solutions by Marxan (black) compared to sites considered by national plans (red hatches); (B) selection frequency from different Marxan runs. DE–Germany, DK–Denmark, PL–Poland, SE–Sweden.

The aim of scenario 4 was to predict the location of suitable sites without considering the costs for connecting offshore wind parks to the electricity grid. Such a scenario can be relevant in the case of the installation of a super-grid linking different wind energy projects or the marked-driven reduction of grid connection costs for distances longer than 50 km. The Marxan settings for scenario 4 are based on the scenario 2 target for energy production (436 km^2^). The results strongly differ from all other scenarios (Fig 7A and 7B). The solution is preferring the areas with the highest wind availability. The largest area selected by Marxan can be found in a relatively shallow part in Danish waters just outside the Natura 2000 area. An additional and previously unselected site can be found in Polish waters at the eastern boundary of the study area.

With a regular occurence of more than 20,000 overwintering long-tailed ducks, Rønne Banke and Adler Grund are both nationally and internationally important bird areas. Long-tailed Ducks can occur up to 30 m water depth [21] For this reason, the Danish Energy Authority made a decision to place wind power sites in areas with water depths of 30 m or deeper [16]. Due to lack of adequate data, it was not possible to represent seabird areas in the Marxan settings in this study and, in consequence, the selected wind power sites partly overlap with this important bird area. Comparing all scenarios, the German site “Arkona-Becken Suedost” is selected as the only area in all cases and can therefore be considered the most suitable site within the study area. The area is situated on the current ferry connection between Sassnitz and Rønne. The ferry connection has the lowest ship traffic intensity in the area that was taken into consideration. If Germany is to establish “Arkona-Becken Suedost” and the neighbouring designated areas in the North, the ferry route would be forced southwards towards the direction of the geographic features Adler Grund and Rønne Banke.

## Discussion and conclusions

For the first time, to our knowledge, Marxan was employed for testing the influence of different energy production targets on the site selection of suitable offshore wind production areas in the Baltic Sea. Selected Marxan simulations were conducted in the Pomeranian Bight / Arkona Basin, including scenarios with and without individual country targets and electricity grid connection costs for a case assuming direct access to a Baltic marine super grid. In our study, only a limited number of targets, conservation, conflict and cost layers were used to demonstrate the potential value of site selection tools in support of an informed MSP decision making. In the context of offshore wind power, Marxan can provide a suitable and systematic evaluation framework for assessing benefits and trade-offs of alternative zoning design and delineation for offshore wind energy development. Since the study was conducted later than the finalization of the national plans, it was not unexpected that the results differ significantly. By increasing the production targets, it could be seen that some of the reservation areas were slightly less suitable with the chosen settings and were chosen in scenario 2 or 3 while other designated areas were never chosen. We found the largest discrepancies between sites selected by Marxan and reserved sites in existing national plans in the Rønne Banke area. Natura 2000 areas were both excluded as suitable sites for offshore wind parks in our Marxan scenarios and the national plans. As mentioned above, the Natura 2000 area at Rønne Banke was first designated in 2010.The result was an overlap between wind park areal reservations and the Natura 2000 area. A later update of the national plan presented after the completion of this study moved the areal reservation to the southeast of the Natura 2000 area [22].

Marxan is not an entirely objective tool. Input parameter choice, model settings, and experimental design depend on the experience of the user (planner and/or modeler) and are based on weighing and prioritizing the different contributing factors. The differences in the solutions between the individual scenarios in this study highlight the sensitivity of Marxan to the definition of targets, target features and conflicting features, as well as cost factors. The quality of an effective decision support tool and subsequent planning process is largely determined by scale and resolution of available ecological and socio-economic data [18, 19, 23]. Such data are often either not available at high resolution or inconsistent, e.g. different data layers have different spatial resolutions.

Especially for birds, the potential conflict with wind mills is high and habitat protection outside the protected areas may be required [24]. This has been taken into consideration in other offshore wind power planning processes and as a result spatial reservation for offshore wind power has been withdrawn in another location in Denmark [25, 26]. Designated Natura 2000 sites were the only ecological feature, which was possible to include in the analysis. Homogeneous data with an adequate resolution on ecological features like seabird populations, marine mammals vulnerable to construction or wind park operations, as well as seabed suitability with impact on construction costs would have been highly desirable, but were impossible to obtain at the time of this study. Thus, the potentially large conflict between seabirds and wind mills could not be detected and resolved by Marxan based on Natura 2000 areas alone.

Simple approaches as demonstrated in this study, still can give valuable information for initial studies and transboundary cases to evaluate how to optimize the production of wind energy if the limitations are clearly communicated. In the future, more complex Marxan setups are desirable in connection with spatial planning of renewable offshore energy projects, where both ecosystem components and cost factors have to be considered. A more detailed determination of individual cost factors might have considerably altered the Marxan predictions in our scenarios. This includes better information on possible access points to the existing electricity grid on land and robust estimates of the possible cost of necessary installation of high voltage transformers in the wind park area. The cost factor depth can be improved by accurate information about seabed stability from surface and lower sediment profiles. Offshore shallow areas like banks are frequently chosen as suitable sites due to the lower costs for small water depths in the Marxan simulations. They often feature sand or coarser sediments at the seabed surface, which might occasionally overlay pockets of unstable substrate affecting the construction costs. Such data was not available at the time of this study, but is an important resource for installing cost efficient foundations for windmills. Another important cost factor not considered in our study is the value of marine ecosystems expressed as the ecosystem services they provide. White et al. [27] developed a framework to use ecosystem service trade-offs in MSP. In a new study, Egli et al [28] developed an approach to systematically incorporate ecosystem services for land based wind power production site selection as costs in Marxan. They showed that decision support tools such as Marxan can help to optimise site selection by mitigating trade-offs between ecosystem services and wind electricity production [28].

## Supporting information

S1 File Supporting data. (ZIP)
